# Iron’s fingerprint of deposits—iron speciation as a geochemical marker

**DOI:** 10.1007/s11356-017-0387-2

**Published:** 2017-10-13

**Authors:** Przemysław Niedzielski, Lidia Kozak

**Affiliations:** Faculty of Chemistry, Department of Analytical Chemistry, Adam Mickiewicz University, 89b Umultowska Street, 61-614 Poznan, Poland

**Keywords:** Iron, Speciation, Flood deposits, Lake and river sediments, Geochemistry

## Abstract

The article describes the studies of the iron speciation (the occurrence of Fe(II), Fe(III), and complexed iron) in acid leachable fraction of deposits. The speciation of iron was diverse for deposits of different origins and was the specific “fingerprint”—marker of deposition processes. The higher occurrence of Fe(II) may be connected with decantation which has been observed for lake sediments, the majority of Fe(III) with upper flow regime erosion-deposition processes, observed for river sediment deposition during flood episodes. As verification for this hypothesis, the explanation for the iron speciation in the different river sediment samples has been used.

**Electronic supplementary material:**

The online version of this article (10.1007/s11356-017-0387-2) contains supplementary material, which is available to authorized users.

## Introduction

The studies on the geochemical markers of sediment/rock origin are important to understand geological processes. The knowledge of “fingerprinting” techniques for sediment source identification using different “fingerprint” properties has been put together in a review paper (Krishnappan et al. 2009) and focused on identification of the sources of sediment transported in river systems. As a “fingerprint” of lake processes, the concentration of the three elements (Al, Fe, K) has been used to identify the source and the deposition patterns of tributary sediments (Righetti et al. 2011). The “chemical fingerprint” as Be, Cr, Fe, and V has been used to identify the source of the origin of arsenic in sediments: geogenic or anthropogenic. The glauconitic sediments have been indicated as a source of arsenic (Barringer et al. 2011). To identify the sediment input and to understand the origin of cave deposits (Mugnano Cave), the combined sedimentological and mineralogical (XRD and SEM) studies of sediments and bedrock have been used (Iacoviello and Martini 2012). The X-ray fluorescence (XRF) technique has been used to study the genetic relationship between mud-rich cave sediments and red surface soils in the Montagnola Senese karst massif, Italy (Iacoviello and Martini 2013). The isotope composition of molybdenum (Chappaz et al. 2012) has been used in the sediment core analysis to distinguish between natural and anthropogenic contributions to aquatic systems, and the use of molybdenum isotopes as “fingerprint” of human impacts has been proposed. The information about the rare earth elements (REEs) occurrence in river sediments (Korea and China) has been used as a potential indicator of the sources of Yellow Sea shelf sediments. The REE “fingerprints” have been used as a tool for tracing the provenance of sediments and for reconstructing the circulation system of the shelf (Xu et al. 2009). The “REE fingerprint” has been used as well for studies of dynamics of the marine sediment (Xiaojing et al. 2010), and sediment transport model has been developed. To recognize metal sources and accumulation processes, the selenium isotopic composition has been determined as the “fingerprint” of the alteration or precipitation processes (Wen and Carignan 2011). The 20 trace element concentration in phosphorites (Bech et al. 2010) has been used as “fingerprint” of the phosphorite origin. The geochemical sediment “fingerprints” has been used to obtain the spatial information on the sediment pathways in the determination of the quantitative budgets of the sediment (D’Haen et al. 2012). The geochemical maps for 44 element concentrations in surface sediments and ratios of elements with similar geochemical behavior (Y/Ho, Nb/Ta, and Zr/Hf) have been used as “fingerprints” of drainage systems in terms of the geochemistry of the upper continental crust (Marx and Kamber 2010). The concentrations of trace metals and the rare earth elements (REEs) in the host rocks, stream sediment, surface waters, and acid mine drainage have been used as “fingerprints” of water–rock interaction (da Silva et al. 2009). For palaeoenvironmental reconstruction, the carbon-to-nitrogen ratios (TOC/TN), Rock-Eval analyses, stable isotope values of bulk nitrogen (d15 N), and organic carbon (d13 Corg) have been used to characterize bulk organic matter (OM) and compared with quantitative microfossil data (Mayr et al. 2009).

The iron determination has been provided both for water (Furman 1962; Watts 1964) and sediment (Cover and Wilhm 1982) samples. The studies focused on the iron occurrence in sediment samples presented in literature for lake sediments (Danen-Louwerse et al. 1993), marine and oceanic sediments (Raiswell and Canfield 1998; Wijsman et al. 2001), coastal sediments (Holmer et al. 1994), and river sediments (Awadallah et al. 1996) frequently were connected with the studies concerning iron relations with other metals (Matijević et al. 2008), for example, arsenic (Endo et al. 2008), phosphorus (Anschutz et al. 1998), manganese (Pakhomova et al. 2007), cerium (Nedel et al. 2010), and with carbon and sulfur (Holmer et al. 1994).

The article describes the studies of the iron speciation (the determination of Fe(II), Fe(III), and complexed iron) in acid leachable fraction of deposits. The speciation of iron was diverse for deposits of different origin and was the specific “fingerprint”—marker of deposition processes.

## Experimental

### Sampling and sample preparation

The study was based on three different groups of samples: lacustrine sediments, floodplain sediments, and river channel deposits. The 50 samples of sediments from different lakes (West Poland) have been obtained from the deepest area of the lakes using the sediment samplers. The 60 samples of flood deposits have been sampled after the flood episodes (Warta river in Poznan, Poland) from the surface layer of deposits (Kozak et al. 2012; Niedzielski et al. 2015b). The samples of river channel deposits have been collected in two ways: for a shallow river (Parseta, Poland), 36 samples were collected in 12 transects crosswise to river way (ca. 40 km along river): at right and left banks and in the middle stream. Additionally, for big river (Vistula, Poland), the 48 sediment samples have been sampled from the middle stream (ca. 50 km along river). All samples have been collected using the plastic tools. The wet samples were subjected to extraction with hydrochloric acid at a concentration of 2 mol L^−1^, according to the previously described procedure (Kozak and Niedzielski 2013). Then, the next parts of samples underwent lyophilization for the purpose of drying (to calculate iron concentration for the dry mass of the samples).

### Analytical procedures for the determination of iron forms

For the study related to forms of iron content in the hydrochloric acid leachable fraction, two analytical procedures developed and published previously were used (Kozak et al. 2013; Niedzielski et al. 2014). The first allowed the simultaneous determination of forms of iron (Fe (II), Fe (III)), complexed iron, and total iron using a tandem combination of UV-Vis spectrometry and atomic absorption spectrometry in a flow injection system. The second method was the spectrophotometric determination of forms of iron using a 2,2′-bipirydyl (for Fe (II)) and ammonium thiocyanate (for Fe (III)) and determination of total iron (Fe) content by atomic absorption spectrometry technique. The factor Fe(II)/Fe(III) has been calculated to compare the differences of the iron speciation in the samples. Additionally, the contents of Ca, Mg, and Mn were determined in sediments and deposit extracts by atomic absorption spectrometry using previously developed methodology (Niedzielski et al. 2015a) and the factors Ca/Mg and Fe/Mn have been calculated. For quality control, the certified standard materials (for total concentration of elements) and standard addition method (for iron speciation) have been used. The statistical analysis has been provided using Statistica 10 software.

## Results and discussion

**Fig. 1:**
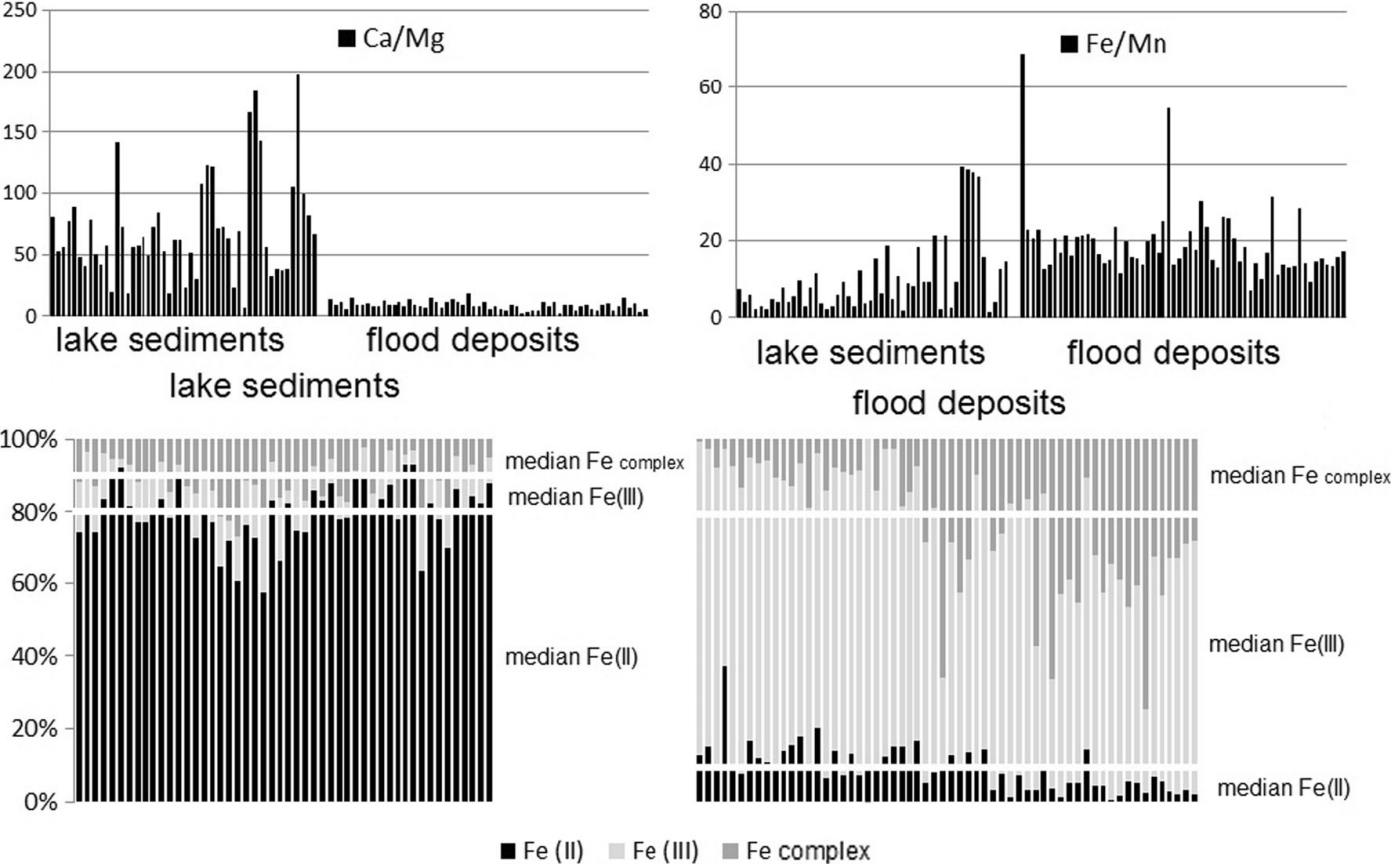
Comparison of calcium, magnesium (as factor Ca/Mg) and iron, manganese (as factor Fe/Mn) occurrence and iron speciation (occurrence of Fe(II), Fe(III), and Fe complexed) in lake sediments (*n* = 50) and flood deposits (*n* = 60) samples

**Fig. 2:**
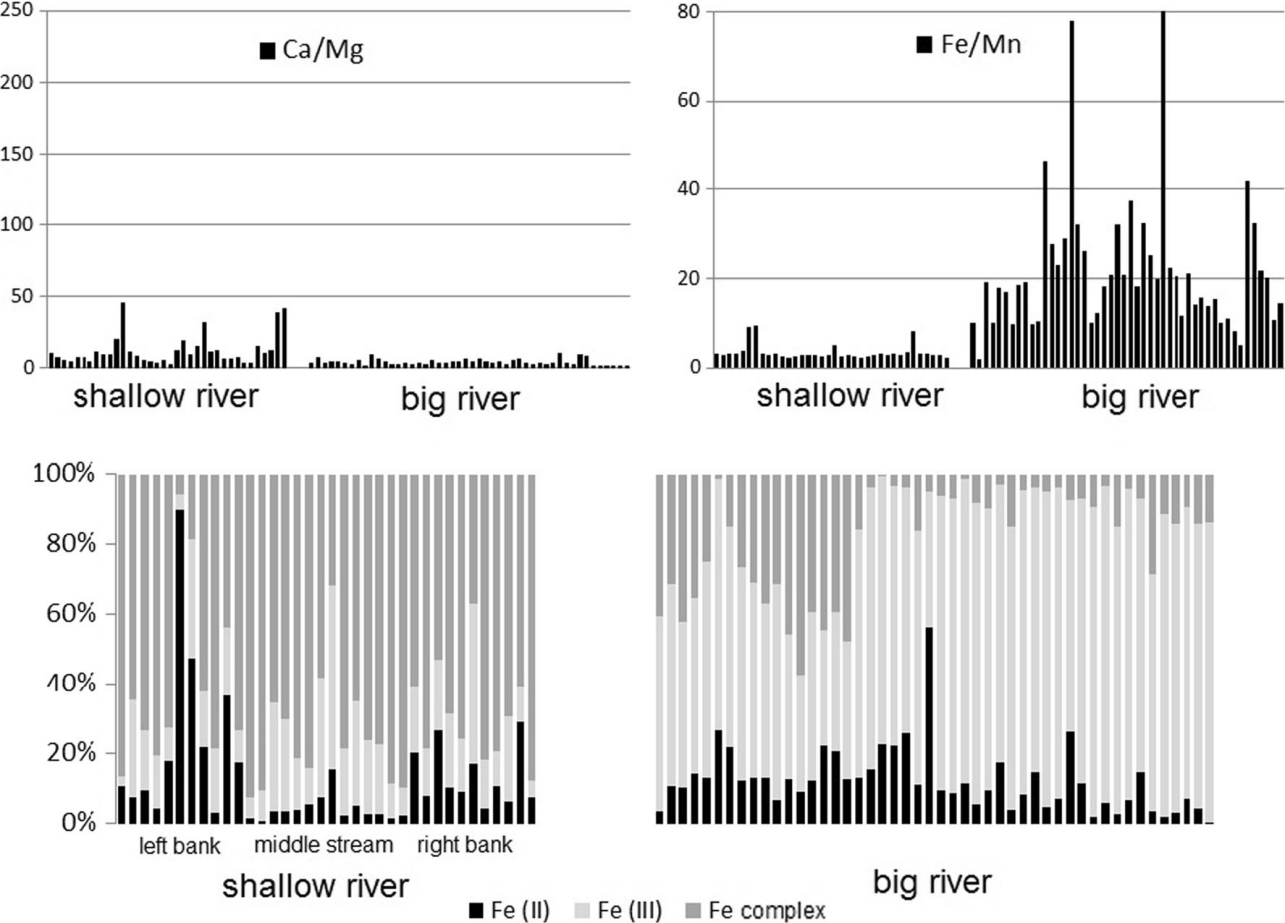
Comparison of calcium, magnesium (as factor Ca/Mg) and iron, manganese (as factor Fe/Mn) occurrence and iron speciation (occurrence of Fe(II), Fe(III), and Fe complexed) in shallow river sediments (*n* = 36) and big river sediments (*n* = 48) samples

The analysis has been provided for 50 lake sediment samples, 60 samples of flood deposits, 36 samples collected in 12 transects of the shallow river, and 48 samples of big river sediments. The samples represented a large lithological variability (coarse-grained the fluvial channel deposits versus the floodplain and lacustrine one). The results of Ca, Mg (as factor Ca/Mg) and Fe, Mn (as factor Fe/Mn) and iron speciation (occurrence of Fe(II), Fe(III), and complexed iron (Fe complex)) in acid leachable fraction have been placed in Figs. 1 and 2.

### Lake sediments and flood deposits

The ways of sedimentation of the lake sediments and flood deposits were different, oversimplifying, in lake depositional environment, the deposit production was decantation process (in the deeper part of lakes, where fluvial clastic inputs are absent) in stable condition (anaerobic in the bottom part of lake); during flood, the deposition processes were short and mainly went in aerobic conditions (Kozak et al. 2012). The character of deposits was different; lake sediments were produced by geological processes and deposited due to fallout processes, while the flood deposits were taken out from the river bed after erosion-deposition processes during the flood episode (Miall 2014). This difference was clearly visible in chemical composition of the deposits: lake sediments contained higher level of calcium than magnesium and higher level of iron than manganese. The difference between the chemical composition of the acid leachable fraction of the lake sediments and flood deposits has been noticeable for iron speciation (Fig. 1). For lake sediments, the dominated form was Fe(II), similarly than for soil environment (Schwertmann 1991)—80% of total iron, the content of the other iron forms was similar: Fe(III)—9% and complexed Fe—11%. The redox potential influences the activity of Fe(II). At the same conditions, the activity of Fe(II) is higher than the Fe(III) compounds (Schwertmann 1991). For flood deposits, the iron speciation has been different: the dominated form was Fe(III)—73% of total iron, the content of Fe(II) was 9%, and Fe complexed constituted 18% of total iron. Following Schwertmann (1991), the oxidation of Fe(II) in waters and its subsequent hydrolysis is a very common process of Fe oxide formation in natural environments (Schwertmann and Murad 1988).

### River sediments

The factors Ca/Mg and Fe/Mn for acid leachable fraction of the big river sediments were close to the factors that characterize the flood deposits; for shallow river, this similarity was not so clear (Fig. 2). The iron speciation in acid leachable fraction of the big river sediments and shallow river sediments collected in the middle stream was closed to the occurrence of iron forms in flood deposits (higher concentration of Fe(III) than Fe(II)) and was different than the occurrence of iron forms of the rest of the shallow river sediments. The deposit production processes in both rivers’ depositional environment were generally different than in lake depositional environment and caused different sediments composition.

**Fig. 3:**
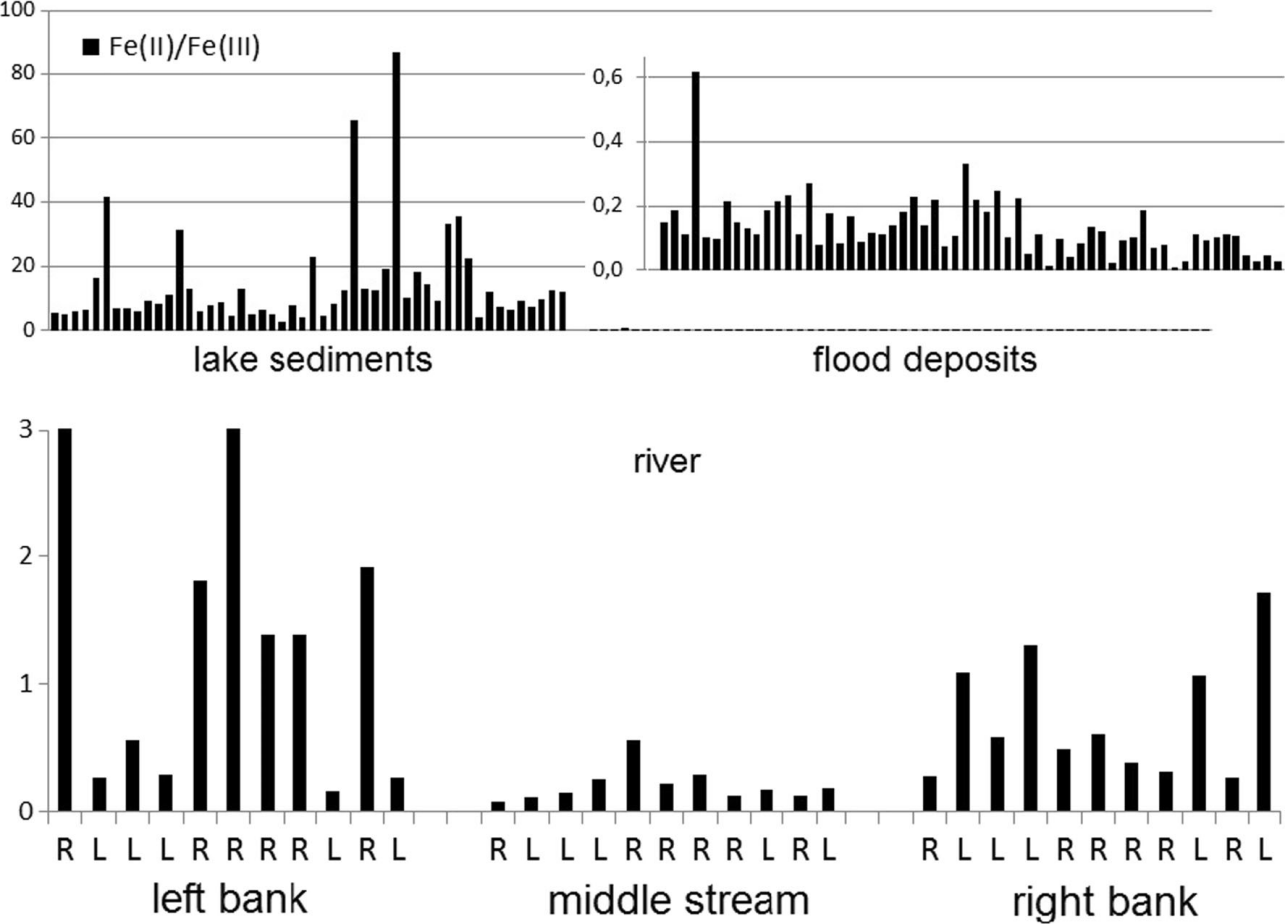
Comparison of the iron speciation (as the factor Fe(II)/Fe(III)) in lake sediments, flood deposits, and the river sediments from the shallow river transects: R—right bend, L—left bend

The general observation (Fig. 3) was the majority of Fe(II)—the factor Fe(II)/Fe(III) values above 1 for decantation process in stable condition of deposition in lake depositional environments and the majority of Fe(III)—the factor Fe(II)/Fe(III) values below 1 for river depositional environments especially for upper flow regime erosion-deposition processes during the flood episode. The higher occurrence of Fe(II) may be connected with decantation, the majority of Fe(III) with upper flow regime erosion-deposition processes. The inorganic reduction of Fe(III) to Fe(II) in deposited sediments is rather slow and the proportion Fe(II)/Fe(III) is stable (White et al. 1991). This hypothesis may be used for the explanation of the different iron speciations in the samples collected at the banks of the shallow river (Fig. 3). For samples collected at the river’s left bank, the dominated iron form was Fe(II)—the factor Fe(II)/Fe(III) values above 1 for the right bends of the river, and the domination of Fe(III)—the factor Fe(II)/Fe(III) values below 1 has been noticed for the left bends of river. For samples collected at the river’s right bank, Fe(II) domination has been noticed for the left bends of the river, and the domination of Fe(III) has been connected with river’s right banks. This observation was with accordance to hypothetical hydrological processes: in the left river bend (L), the upper flow regime erosion/deposition processes were dominated for the left bank, and the lower flow regime deposition was dominated for the right bank; in the right bend (R), the erosion-deposition processes were reversed: upper flow regime erosion/deposition for the right bank and lower flow regime deposition for the left bank (Fig. 3).

### Statistical analysis

**Fig. 4:**
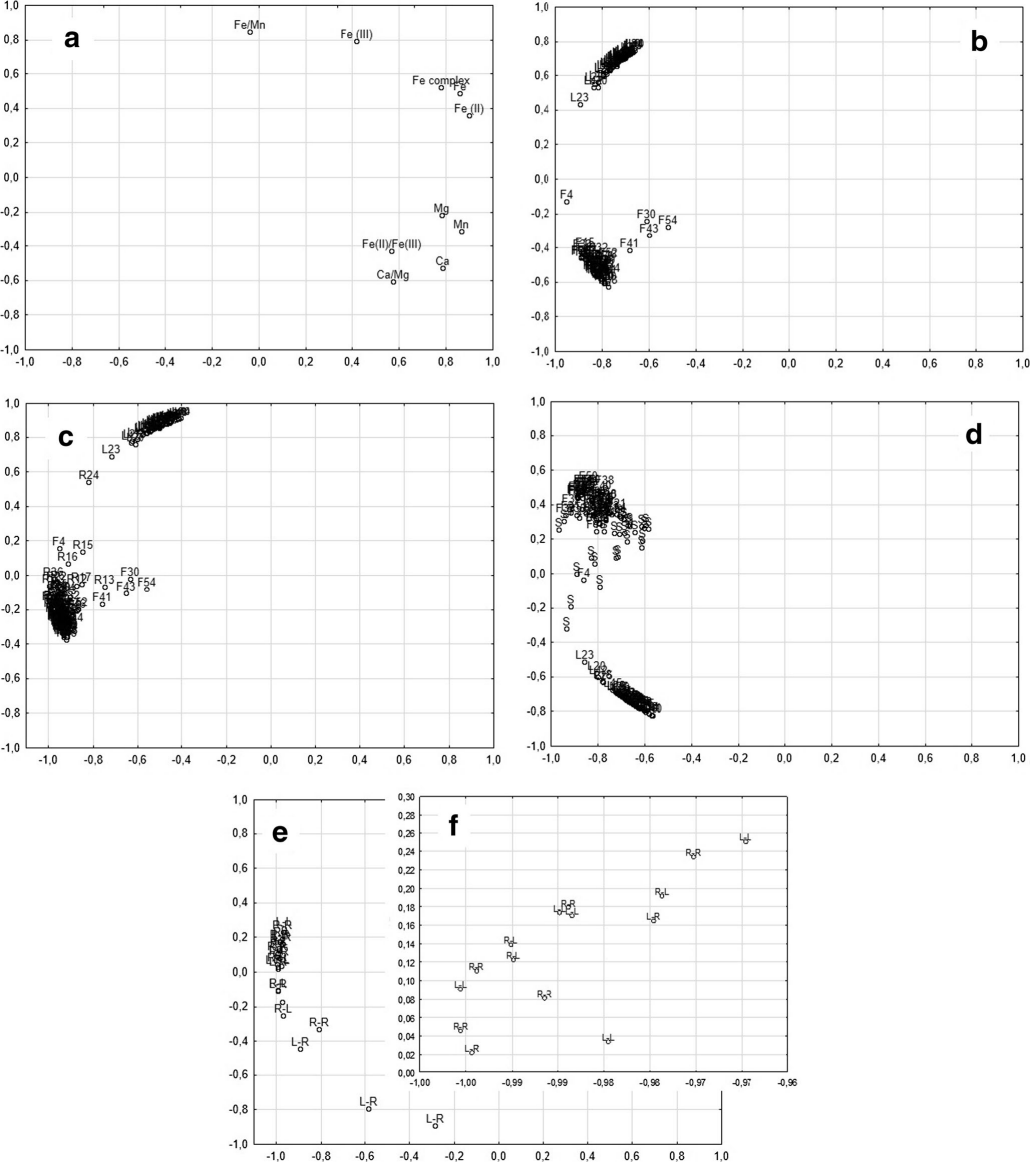
For the results of exploration analysis—factor analysis, the number of components was 2. **a** The analysis of parameters. **b** The analysis of the iron speciation in the samples of lake sediments (L, *n* = 50) and flood deposits (F, *n* = 60). **c** The analysis of the iron speciation in the samples of lake sediments (L, *n* = 50), flood deposits (F, *n* = 60), and big river sediments (R, *n* = 48). **d** The analysis of the iron speciation in the samples of lake sediments (L, *n* = 50), flood deposits (F, *n* = 60), and shallow river sediments from transects (S, *n* = 36). **e**–**f**. The analysis of the iron speciation in the samples of shallow river sediments from transects (*n* = 36). L-L left river bank, the bend to left; L-R left river bank, the bend to left; R-L right river bank, the bend to left; R-R right river bank, the bend to left

Additionally, the statistical analysis—the exploratory factor analysis (FA) has been performed (Fig. 4). The FA results for occurrence of determined parameters and factors calculated (Fig. 4a) showed that the shaping of Fe(III) concentration was different than Fe(II) and different than Fe complex, Fe, Ca, Mg, and Mn concentrations. The FA (Fig. 4b) for iron occurrence and speciation (the total content of Fe and occurrence of Fe(II), Fe(III), and Fe complex) in acid leachable fraction of lake sediments and flood deposits divided the results for two separate groups: for lake sediments and for flood deposits. The same analysis provided additionally for big river (Fig. 4c) and shallow river (Fig. 4d) samples showed that most of the river samples demonstrated the similarity of iron speciation to flood deposits. However, for the number of samples from the shallow river, the similarity was not so clear. The FA (Fig. 4e and in different scale Fig. 4f) provided for iron occurrence and speciation in acid leachable fraction of shallow river sediments shoved the highest difference of iron speciation for samples collected from the left river bank at the right river bends (L-R). This statement is in accordance with the abovementioned results.

## Conclusions

The results of iron speciation studies (the determination of Fe(II), Fe(III), and complexed iron) in the acid leachable fraction may be used as the marker (“fingerprint”) of the character of the depositional processes. The studies on the iron speciation in the lake sediments, river sediments, and flood deposits showed the different iron speciation patterns for different depositional environments. The decantation as in the lake depositional environment produces the majority of Fe(II), and the upper flow regime erosion-sedimentation processes like the river’s flood deposition caused the majority of Fe(III). The knowledge about iron speciation gives the opportunity to decide about the character of deposition/erosion processes based on chemical composition of the deposits.

## Electronic supplementary material

ESM 1 (DOC 289 kb)
