# Supplementary material for: Iron’s fingerprint of deposits—iron speciation as a geochemical marker
Source: Environ Sci Pollut Res Int. 2017 Oct 13;25(1):242–8. doi: 10.1007/s11356-017-0387-2 (PMC5756553; doi:10.1007/s11356-017-0387-2)
Supplement: Supplementary file 1 — (DOC 289 kb) [file 11356_2017_387_MOESM1_ESM.doc]

**Iron’s fingerprint of deposits – iron speciation as a geochemical marker**

**Przemysław Niedzielski, Lidia Kozak**

Supplementary data

| **g kg-1** | **Fe (II)** | **Fe (III)** | **Fe complex** | **Fe** | **Ca** | **Mg** | **Mn** | **Ca/Mg** | **Fe/Mn** | **Fe(II)/Fe(III)** |
| --- | --- | --- | --- | --- | --- | --- | --- | --- | --- | --- |
| **lake sediments** | |  |  |  |  |  |  |  |  |  |
| **L1** | 3,72 | 0,68 | 0,60 | 5,01 | 300 | 3,70 | 0,66 | 81,1 | 7,6 | 5,47 |
| **L2** | 1,82 | 0,36 | 0,08 | 2,26 | 225 | 4,32 | 0,54 | 52,1 | 4,2 | 5,06 |
| **L3** | 3,91 | 0,66 | 0,69 | 5,25 | 188 | 3,38 | 0,89 | 55,6 | 5,9 | 5,92 |
| **L4** | 1,87 | 0,28 | 0,09 | 2,24 | 209 | 2,70 | 1,00 | 77,4 | 2,2 | 6,68 |
| **L5** | 1,62 | 0,10 | 0,10 | 1,82 | 251 | 2,81 | 0,62 | 89,3 | 2,9 | 16,20 |
| **L6** | 1,67 | 0,04 | 0,10 | 1,81 | 226 | 4,70 | 0,85 | 48,1 | 2,1 | 41,75 |
| **L7** | 3,19 | 0,45 | 0,28 | 3,92 | 238 | 5,90 | 0,82 | 40,3 | 4,8 | 7,09 |
| **L8** | 2,00 | 0,29 | 0,31 | 2,60 | 237 | 3,05 | 0,64 | 77,7 | 4,1 | 6,90 |
| **L9** | 2,89 | 0,50 | 0,35 | 3,73 | 204 | 4,07 | 0,49 | 50,1 | 7,6 | 5,78 |
| **L10** | 4,94 | 0,53 | 0,62 | 6,09 | 162 | 3,83 | 1,51 | 42,3 | 4,0 | 9,32 |
| **L11** | 2,07 | 0,25 | 0,16 | 2,48 | 222 | 3,90 | 0,45 | 56,9 | 5,5 | 8,28 |
| **L12** | 5,53 | 0,50 | 1,04 | 7,08 | 71 | 3,69 | 0,72 | 19,1 | 9,8 | 11,06 |
| **L13** | 3,74 | 0,12 | 0,29 | 4,15 | 179 | 1,26 | 1,37 | 142,1 | 3,0 | 31,17 |
| **L14** | 3,76 | 0,29 | 0,61 | 4,66 | 237 | 3,30 | 0,61 | 71,8 | 7,6 | 12,97 |
| **L15** | 5,61 | 0,96 | 1,16 | 7,73 | 121 | 6,56 | 0,67 | 18,4 | 11,5 | 5,84 |
| **L16** | 2,67 | 0,33 | 0,29 | 3,29 | 249 | 4,47 | 0,87 | 55,7 | 3,8 | 8,09 |
| **L17** | 2,79 | 0,31 | 0,52 | 3,63 | 189 | 3,29 | 1,66 | 57,4 | 2,2 | 9,00 |
| **L18** | 1,91 | 0,40 | 0,63 | 2,94 | 203 | 3,16 | 0,97 | 64,2 | 3,0 | 4,78 |
| **L19** | 2,84 | 0,22 | 0,89 | 3,95 | 186 | 3,81 | 0,66 | 48,8 | 6,0 | 12,91 |
| **L20** | 3,04 | 0,63 | 1,35 | 5,02 | 225 | 3,10 | 0,55 | 72,6 | 9,1 | 4,83 |
| **L21** | 2,92 | 0,46 | 0,44 | 3,82 | 103 | 1,22 | 0,68 | 84,4 | 5,6 | 6,35 |
| **L22** | 2,92 | 0,58 | 0,51 | 4,01 | 208 | 3,93 | 1,45 | 52,9 | 2,8 | 5,03 |
| **L23** | 1,50 | 0,58 | 0,53 | 2,61 | 33 | 1,81 | 0,21 | 18,3 | 12,4 | 2,59 |
| **L24** | 1,63 | 0,21 | 0,12 | 1,97 | 285 | 4,61 | 0,55 | 61,8 | 3,6 | 7,76 |
| **L25** | 4,77 | 1,22 | 1,17 | 7,16 | 197 | 3,20 | 1,61 | 61,6 | 4,4 | 3,91 |
| **L26** | 8,97 | 0,39 | 1,55 | 10,90 | 136 | 5,92 | 0,72 | 23,0 | 15,1 | 23,00 |
| **L27** | 4,27 | 0,93 | 0,52 | 5,72 | 182 | 3,58 | 0,93 | 50,8 | 6,2 | 4,59 |
| **L28** | 5,65 | 0,66 | 1,28 | 7,59 | 133 | 4,46 | 0,41 | 29,8 | 18,5 | 8,56 |
| **L29** | 2,77 | 0,22 | 0,24 | 3,24 | 274 | 2,54 | 0,70 | 107,9 | 4,6 | 12,59 |
| **L30** | 21,0 | 0,32 | 3,94 | 25,20 | 262 | 2,14 | 2,32 | 122,4 | 10,9 | 65,63 |
| **L31** | 1,31 | 0,10 | 0,08 | 1,50 | 179 | 1,47 | 0,89 | 121,8 | 1,7 | 13,10 |
| **L32** | 4,12 | 0,33 | 0,83 | 5,28 | 136 | 1,92 | 0,58 | 70,8 | 9,1 | 12,48 |
| **L33** | 9,60 | 0,50 | 2,15 | 12,30 | 257 | 3,57 | 1,51 | 72,0 | 8,1 | 19,20 |
| **L34** | 11,23 | 0,13 | 1,09 | 12,50 | 226 | 3,59 | 0,68 | 63,0 | 18,4 | 86,38 |
| **L35** | 4,55 | 0,44 | 0,13 | 5,11 | 98 | 4,27 | 0,54 | 22,9 | 9,5 | 10,34 |
| **L36** | 12,6 | 0,69 | 2,33 | 15,60 | 239 | 3,45 | 1,68 | 69,3 | 9,3 | 18,26 |
| **L37** | 0,71 | 0,05 | 0,09 | 0,85 | 1 | 0,19 | 0,04 | 6,3 | 21,3 | 14,20 |
| **L38** | 1,60 | 0,17 | 0,06 | 1,83 | 191 | 1,15 | 0,90 | 166,1 | 2,0 | 9,41 |
| **L39** | 20,1 | 0,61 | 5,16 | 25,80 | 259 | 1,41 | 1,22 | 183,7 | 21,1 | 32,95 |
| **L40** | 2,49 | 0,07 | 0,12 | 2,68 | 260 | 1,82 | 1,02 | 142,9 | 2,6 | 35,57 |
| **L41** | 6,44 | 0,29 | 0,21 | 6,94 | 63 | 1,12 | 0,74 | 56,0 | 9,4 | 22,21 |
| **L42** | 50,9 | 12,70 | 16,83 | 80,44 | 218 | 6,67 | 2,05 | 32,7 | 39,1 | 4,01 |
| **L43** | 28,9 | 2,40 | 3,76 | 35,05 | 211 | 5,45 | 0,91 | 38,8 | 38,5 | 12,05 |
| **L44** | 28,0 | 3,78 | 4,08 | 35,83 | 184 | 4,97 | 0,95 | 36,9 | 37,7 | 7,40 |
| **L45** | 19,3 | 3,00 | 5,34 | 27,68 | 188 | 4,89 | 0,75 | 38,3 | 36,8 | 6,45 |
| **L46** | 3,13 | 0,34 | 0,17 | 3,64 | 100 | 0,95 | 0,23 | 105,3 | 15,8 | 9,30 |
| **L47** | 0,73 | 0,10 | 0,09 | 0,92 | 244 | 1,23 | 0,62 | 197,6 | 1,5 | 7,45 |
| **L48** | 3,33 | 0,34 | 0,29 | 3,95 | 141 | 1,41 | 0,93 | 99,4 | 4,2 | 9,92 |
| **L49** | 4,81 | 0,39 | 0,64 | 5,84 | 215 | 2,64 | 0,47 | 81,6 | 12,5 | 12,38 |
| **L50** | 5,61 | 0,46 | 0,32 | 6,39 | 187 | 2,83 | 0,44 | 66,0 | 14,7 | 12,31 |
| **flood deposits** | |  |  |  |  |  |  |  |  |  |
| **F1** | 1,51 | 10,14 | 0,11 | 11,76 | 9,23 | 0,67 | 0,17 | 13,8 | 68,7 | 0,15 |
| **F2** | 0,19 | 1,04 | 0,04 | 1,27 | 1,77 | 0,19 | 0,06 | 9,5 | 22,8 | 0,18 |
| **F3** | 0,09 | 0,82 | 0,08 | 0,99 | 1,53 | 0,13 | 0,05 | 12,0 | 20,6 | 0,11 |
| **F4** | 1,41 | 2,28 | 0,10 | 3,79 | 2,74 | 0,47 | 0,17 | 5,9 | 23,0 | 0,62 |
| **F5** | 0,13 | 1,24 | 0,11 | 1,48 | 2,48 | 0,17 | 0,12 | 14,8 | 12,5 | 0,10 |
| **F6** | 0,05 | 0,49 | 0,08 | 0,62 | 1,11 | 0,12 | 0,04 | 9,6 | 13,9 | 0,10 |
| **F7** | 0,15 | 0,71 | 0,05 | 0,91 | 1,58 | 0,18 | 0,04 | 8,9 | 20,4 | 0,22 |
| **F8** | 0,09 | 0,60 | 0,05 | 0,74 | 1,25 | 0,12 | 0,04 | 10,1 | 16,8 | 0,15 |
| **F9** | 0,09 | 0,66 | 0,05 | 0,79 | 0,88 | 0,11 | 0,04 | 8,3 | 21,3 | 0,13 |
| **F10** | 0,09 | 0,79 | 0,10 | 0,98 | 1,11 | 0,13 | 0,06 | 8,5 | 16,1 | 0,11 |
| **F11** | 0,13 | 0,71 | 0,11 | 0,95 | 1,45 | 0,11 | 0,05 | 13,2 | 21,0 | 0,18 |
| **F12** | 0,13 | 0,59 | 0,11 | 0,82 | 0,90 | 0,10 | 0,04 | 9,3 | 21,2 | 0,21 |
| **F13** | 0,25 | 1,08 | 0,09 | 1,43 | 1,14 | 0,13 | 0,07 | 9,1 | 21,7 | 0,24 |
| **F14** | 0,09 | 0,84 | 0,22 | 1,15 | 1,50 | 0,14 | 0,06 | 11,0 | 20,5 | 0,11 |
| **F15** | 0,32 | 1,20 | 0,06 | 1,59 | 1,05 | 0,14 | 0,10 | 7,7 | 16,5 | 0,27 |
| **F16** | 0,05 | 0,58 | 0,11 | 0,73 | 1,30 | 0,09 | 0,05 | 13,7 | 14,0 | 0,08 |
| **F17** | 0,10 | 0,56 | 0,06 | 0,71 | 1,21 | 0,13 | 0,05 | 9,5 | 14,9 | 0,18 |
| **F18** | 0,08 | 0,91 | 0,10 | 1,08 | 0,92 | 0,12 | 0,05 | 7,6 | 23,5 | 0,08 |
| **F19** | 0,12 | 0,71 | 0,09 | 0,92 | 0,93 | 0,13 | 0,08 | 6,9 | 11,6 | 0,17 |
| **F20** | 0,05 | 0,61 | 0,06 | 0,72 | 1,18 | 0,08 | 0,04 | 14,6 | 19,6 | 0,09 |
| **F21** | 0,08 | 0,67 | 0,00 | 0,75 | 1,24 | 0,11 | 0,05 | 11,4 | 15,5 | 0,12 |
| **F22** | 0,06 | 0,51 | 0,10 | 0,66 | 0,72 | 0,10 | 0,04 | 7,2 | 15,2 | 0,11 |
| **F23** | 0,10 | 0,67 | 0,02 | 0,79 | 1,05 | 0,09 | 0,06 | 11,2 | 13,7 | 0,14 |
| **F24** | 0,12 | 0,66 | 0,02 | 0,80 | 1,72 | 0,13 | 0,04 | 13,3 | 19,7 | 0,18 |
| **F25** | 0,20 | 0,86 | 0,24 | 1,30 | 1,90 | 0,17 | 0,06 | 11,2 | 21,8 | 0,23 |
| **F26** | 0,09 | 0,63 | 0,12 | 0,84 | 1,16 | 0,12 | 0,05 | 9,6 | 17,0 | 0,14 |
| **F27** | 0,16 | 0,72 | 0,07 | 0,95 | 2,62 | 0,15 | 0,04 | 18,1 | 25,0 | 0,22 |
| **F28** | 0,09 | 1,13 | 0,49 | 1,70 | 0,84 | 0,11 | 0,03 | 8,0 | 54,7 | 0,08 |
| **F29** | 0,26 | 2,44 | 0,63 | 3,33 | 2,42 | 0,32 | 0,24 | 7,6 | 13,9 | 0,11 |
| **F30** | 0,30 | 0,91 | 2,37 | 3,59 | 5,68 | 0,48 | 0,23 | 11,9 | 15,4 | 0,33 |
| **F31** | 0,27 | 1,24 | 0,60 | 2,11 | 1,40 | 0,28 | 0,12 | 5,0 | 18,1 | 0,22 |
| **F32** | 0,11 | 0,60 | 0,53 | 1,24 | 2,06 | 0,27 | 0,06 | 7,6 | 22,4 | 0,18 |
| **F33** | 0,29 | 1,18 | 0,73 | 2,21 | 1,11 | 0,19 | 0,12 | 6,0 | 17,7 | 0,25 |
| **F34** | 0,19 | 1,80 | 0,22 | 2,21 | 1,14 | 0,27 | 0,07 | 4,2 | 30,3 | 0,10 |
| **F35** | 0,58 | 2,59 | 0,87 | 4,04 | 4,33 | 0,47 | 0,17 | 9,3 | 23,4 | 0,22 |
| **F36** | 0,04 | 0,86 | 0,40 | 1,31 | 0,69 | 0,09 | 0,09 | 7,5 | 15,0 | 0,05 |
| **F37** | 0,04 | 0,37 | 0,15 | 0,56 | 0,25 | 0,13 | 0,04 | 2,0 | 12,9 | 0,11 |
| **F38** | 0,04 | 2,91 | 0,64 | 3,59 | 1,36 | 0,38 | 0,14 | 3,5 | 26,4 | 0,01 |
| **F39** | 0,08 | 0,81 | 0,22 | 1,11 | 0,43 | 0,11 | 0,04 | 3,9 | 25,9 | 0,10 |
| **F40** | 0,09 | 2,21 | 0,46 | 2,76 | 1,18 | 0,26 | 0,13 | 4,5 | 20,6 | 0,04 |
| **F41** | 0,03 | 0,32 | 0,47 | 0,81 | 1,78 | 0,15 | 0,06 | 11,9 | 14,5 | 0,08 |
| **F42** | 0,12 | 0,84 | 0,17 | 1,12 | 1,19 | 0,15 | 0,06 | 7,8 | 18,5 | 0,14 |
| **F43** | 0,04 | 0,34 | 0,76 | 1,14 | 1,97 | 0,18 | 0,16 | 11,2 | 7,2 | 0,12 |
| **F44** | 0,01 | 0,67 | 0,52 | 1,20 | 0,38 | 0,19 | 0,08 | 2,0 | 14,3 | 0,02 |
| **F45** | 0,05 | 0,51 | 0,36 | 0,92 | 0,83 | 0,09 | 0,09 | 9,0 | 9,9 | 0,09 |
| **F46** | 0,04 | 0,35 | 0,32 | 0,71 | 1,25 | 0,13 | 0,04 | 9,4 | 17,0 | 0,10 |
| **F47** | 0,07 | 0,38 | 0,05 | 0,51 | 0,46 | 0,10 | 0,02 | 4,4 | 31,6 | 0,19 |
| **F48** | 0,03 | 0,37 | 0,19 | 0,58 | 0,82 | 0,11 | 0,05 | 7,5 | 11,2 | 0,07 |
| **F49** | 0,05 | 0,58 | 0,47 | 1,09 | 1,24 | 0,14 | 0,08 | 8,9 | 13,8 | 0,08 |
| **F50** | 0,00 | 0,63 | 0,33 | 0,97 | 0,71 | 0,14 | 0,07 | 5,1 | 13,0 | 0,01 |
| **F51** | 0,02 | 0,56 | 0,37 | 0,95 | 1,13 | 0,24 | 0,07 | 4,8 | 13,6 | 0,03 |
| **F52** | 0,05 | 0,43 | 0,42 | 0,90 | 0,87 | 0,10 | 0,03 | 8,8 | 28,3 | 0,11 |
| **F53** | 0,05 | 0,49 | 0,37 | 0,90 | 0,72 | 0,07 | 0,06 | 10,4 | 14,3 | 0,09 |
| **F54** | 0,03 | 0,26 | 0,84 | 1,12 | 0,45 | 0,09 | 0,12 | 4,8 | 9,4 | 0,10 |
| **F55** | 0,05 | 0,45 | 0,24 | 0,74 | 0,49 | 0,06 | 0,05 | 8,2 | 14,7 | 0,11 |
| **F56** | 0,09 | 0,80 | 0,68 | 1,56 | 1,17 | 0,08 | 0,10 | 15,0 | 15,4 | 0,11 |
| **F57** | 0,02 | 0,52 | 0,27 | 0,81 | 0,60 | 0,10 | 0,06 | 6,3 | 13,7 | 0,05 |
| **F58** | 0,02 | 0,57 | 0,28 | 0,87 | 0,89 | 0,09 | 0,06 | 10,0 | 13,5 | 0,03 |
| **F59** | 0,03 | 0,73 | 0,31 | 1,07 | 0,40 | 0,14 | 0,07 | 2,8 | 15,6 | 0,04 |
| **F60** | 0,03 | 1,04 | 0,41 | 1,48 | 0,95 | 0,18 | 0,09 | 5,3 | 17,2 | 0,03 |
| **big river sediments** | | |  |  |  |  |  |  |  |  |
| **R1** | 0,02 | 0,33 | 0,24 | 0,59 | 0,48 | 0,15 | 0,06 | 3,2 | 10,2 | 0,06 |
| **R2** | 0,08 | 0,41 | 0,22 | 0,71 | 0,86 | 0,11 | 0,36 | 7,8 | 2,0 | 0,19 |
| **R3** | 0,49 | 2,23 | 1,99 | 4,70 | 2,61 | 0,80 | 0,24 | 3,2 | 19,3 | 0,22 |
| **R4** | 0,28 | 0,98 | 0,69 | 1,94 | 1,99 | 0,43 | 0,19 | 4,6 | 10,0 | 0,28 |
| **R5** | 0,24 | 1,15 | 0,46 | 1,85 | 1,84 | 0,41 | 0,10 | 4,5 | 18,1 | 0,21 |
| **R6** | 0,92 | 2,48 | 0,05 | 3,44 | 3,17 | 0,94 | 0,20 | 3,4 | 17,1 | 0,37 |
| **R7** | 0,15 | 0,42 | 0,10 | 0,67 | 0,36 | 0,13 | 0,07 | 2,9 | 9,7 | 0,35 |
| **R8** | 0,13 | 0,64 | 0,28 | 1,05 | 0,81 | 0,14 | 0,06 | 5,8 | 18,7 | 0,20 |
| **R9** | 0,10 | 0,43 | 0,24 | 0,77 | 0,35 | 0,22 | 0,04 | 1,6 | 19,3 | 0,23 |
| **R10** | 0,19 | 0,73 | 0,54 | 1,46 | 4,74 | 0,49 | 0,15 | 9,8 | 9,9 | 0,26 |
| **R11** | 0,03 | 0,31 | 0,16 | 0,50 | 0,83 | 0,13 | 0,05 | 6,4 | 10,5 | 0,11 |
| **R12** | 0,22 | 0,73 | 0,80 | 1,75 | 2,27 | 0,56 | 0,04 | 4,0 | 46,6 | 0,31 |
| **R13** | 0,14 | 0,53 | 0,89 | 1,56 | 1,12 | 0,42 | 0,06 | 2,7 | 27,7 | 0,27 |
| **R14** | 0,17 | 0,66 | 0,54 | 1,36 | 0,93 | 0,31 | 0,06 | 3,0 | 22,9 | 0,25 |
| **R15** | 0,20 | 0,30 | 0,41 | 0,91 | 0,76 | 0,20 | 0,03 | 3,8 | 28,9 | 0,68 |
| **R16** | 0,59 | 1,14 | 1,12 | 2,85 | 1,52 | 0,54 | 0,04 | 2,8 | 78,0 | 0,52 |
| **R17** | 0,21 | 0,65 | 0,77 | 1,63 | 1,33 | 0,39 | 0,05 | 3,5 | 32,1 | 0,32 |
| **R18** | 0,32 | 1,69 | 0,37 | 2,38 | 1,51 | 0,50 | 0,09 | 3,0 | 26,3 | 0,19 |
| **R19** | 0,68 | 3,56 | 0,17 | 4,41 | 7,33 | 1,33 | 0,43 | 5,5 | 10,2 | 0,19 |
| **R20** | 1,51 | 5,05 | 0,03 | 6,59 | 8,51 | 2,14 | 0,53 | 4,0 | 12,3 | 0,30 |
| **R21** | 1,13 | 3,72 | 0,16 | 5,01 | 5,16 | 1,53 | 0,28 | 3,4 | 18,2 | 0,30 |
| **R22** | 1,50 | 4,10 | 0,21 | 5,81 | 6,13 | 1,27 | 0,28 | 4,8 | 20,9 | 0,37 |
| **R23** | 0,19 | 1,21 | 0,27 | 1,67 | 1,63 | 0,35 | 0,05 | 4,6 | 32,3 | 0,16 |
| **R24** | 1,77 | 1,22 | 0,15 | 3,14 | 7,33 | 1,21 | 0,15 | 6,1 | 20,7 | 1,45 |
| **R25** | 0,20 | 1,78 | 0,13 | 2,11 | 1,97 | 0,41 | 0,06 | 4,8 | 37,3 | 0,11 |
| **R26** | 0,13 | 1,24 | 0,10 | 1,47 | 1,86 | 0,31 | 0,08 | 6,1 | 18,2 | 0,11 |
| **R27** | 0,21 | 1,54 | 0,03 | 1,77 | 1,49 | 0,35 | 0,05 | 4,2 | 32,5 | 0,13 |
| **R28** | 0,30 | 4,67 | 0,44 | 5,41 | 3,30 | 0,87 | 0,21 | 3,8 | 25,3 | 0,06 |
| **R29** | 0,15 | 1,27 | 0,16 | 1,57 | 1,40 | 0,32 | 0,08 | 4,4 | 19,9 | 0,12 |
| **R30** | 0,81 | 3,73 | 0,13 | 4,67 | 2,08 | 0,74 | 0,05 | 2,8 | 86,1 | 0,22 |
| **R31** | 0,08 | 1,56 | 0,28 | 1,92 | 3,21 | 0,59 | 0,09 | 5,5 | 22,3 | 0,05 |
| **R32** | 0,16 | 1,62 | 0,08 | 1,86 | 3,15 | 0,51 | 0,09 | 6,1 | 20,5 | 0,10 |
| **R33** | 0,82 | 4,50 | 0,20 | 5,52 | 6,30 | 1,98 | 0,48 | 3,2 | 11,5 | 0,18 |
| **R34** | 0,20 | 3,76 | 0,20 | 4,16 | 2,36 | 0,85 | 0,20 | 2,8 | 21,0 | 0,05 |
| **R35** | 0,89 | 10,74 | 0,45 | 12,08 | 7,40 | 2,34 | 0,85 | 3,2 | 14,2 | 0,08 |
| **R36** | 1,60 | 4,05 | 0,45 | 6,10 | 4,64 | 1,77 | 0,39 | 2,6 | 15,8 | 0,40 |
| **R37** | 0,60 | 4,30 | 0,38 | 5,28 | 3,83 | 1,26 | 0,38 | 3,0 | 14,0 | 0,14 |
| **R38** | 0,02 | 1,09 | 0,11 | 1,23 | 1,23 | 0,12 | 0,08 | 9,9 | 15,3 | 0,02 |
| **R39** | 0,12 | 1,78 | 0,06 | 1,96 | 1,22 | 0,34 | 0,19 | 3,6 | 10,1 | 0,07 |
| **R40** | 0,06 | 2,03 | 0,37 | 2,46 | 0,88 | 0,40 | 0,22 | 2,2 | 11,2 | 0,03 |
| **R41** | 0,17 | 2,25 | 0,10 | 2,52 | 2,01 | 0,23 | 0,31 | 8,9 | 8,1 | 0,08 |
| **R42** | 0,73 | 3,84 | 0,33 | 4,91 | 6,85 | 0,85 | 0,98 | 8,1 | 5,0 | 0,19 |
| **R43** | 0,07 | 1,34 | 0,57 | 1,98 | 0,35 | 0,18 | 0,05 | 1,9 | 41,8 | 0,05 |
| **R44** | 0,01 | 0,54 | 0,07 | 0,62 | 0,30 | 0,18 | 0,02 | 1,7 | 32,5 | 0,02 |
| **R45** | 0,03 | 0,72 | 0,12 | 0,87 | 0,26 | 0,20 | 0,04 | 1,3 | 21,8 | 0,04 |
| **R46** | 0,03 | 0,39 | 0,04 | 0,47 | 0,21 | 0,17 | 0,02 | 1,2 | 20,3 | 0,09 |
| **R47** | 0,03 | 0,56 | 0,10 | 0,69 | 0,37 | 0,18 | 0,06 | 2,0 | 10,9 | 0,06 |
| **R48** | 0,00 | 0,59 | 0,09 | 0,69 | 0,30 | 0,26 | 0,05 | 1,2 | 14,6 | 0,00 |
| **shallow river sediments** | | |  |  |  |  |  |  |  |  |
| **1 L** | 1,43 | 0,40 | 11,47 | 13,3 | 6,61 | 4,19 | 0,65 | 10,2 | 3,2 | 3,57 |
| **2 L** | 0,50 | 1,90 | 4,34 | 6,74 | 4,26 | 2,39 | 0,59 | 7,2 | 2,8 | 0,26 |
| **3 L** | 0,65 | 1,15 | 4,95 | 6,74 | 3,93 | 2,06 | 0,72 | 5,5 | 3,3 | 0,56 |
| **4 L** | 0,28 | 0,95 | 5,07 | 6,30 | 3,87 | 1,94 | 0,90 | 4,3 | 3,2 | 0,29 |
| **5 L** | 1,70 | 0,94 | 6,90 | 9,54 | 5,38 | 2,52 | 0,77 | 7,0 | 3,8 | 1,81 |
| **6 L** | 24,9 | 1,21 | 1,55 | 27,7 | 13,8 | 2,98 | 1,81 | 7,6 | 9,3 | 20,61 |
| **7 L** | 1,76 | 1,27 | 0,68 | 3,71 | 3,61 | 0,39 | 0,78 | 4,6 | 9,4 | 1,38 |
| **8 L** | 4,35 | 3,15 | 12,20 | 19,7 | 7,13 | 6,11 | 0,62 | 11,6 | 3,2 | 1,38 |
| **9 L** | 0,54 | 3,30 | 13,96 | 17,8 | 6,36 | 6,18 | 0,72 | 8,9 | 2,9 | 0,16 |
| **10 L** | 7,61 | 3,96 | 9,03 | 20,6 | 8,74 | 6,64 | 0,96 | 9,1 | 3,1 | 1,92 |
| **11 L** | 3,03 | 1,63 | 12,74 | 17,4 | 5,54 | 6,65 | 0,28 | 20,1 | 2,6 | 1,86 |
| **12 L** | 0,23 | 0,87 | 13,10 | 14,2 | 9,75 | 6,10 | 0,22 | 45,3 | 2,3 | 0,27 |
| **1 M** | 0,09 | 1,11 | 11,50 | 12,7 | 6,19 | 4,82 | 0,54 | 11,4 | 2,6 | 0,08 |
| **2 M** | 0,26 | 2,21 | 4,63 | 7,10 | 4,11 | 2,38 | 0,51 | 8,0 | 3,0 | 0,12 |
| **3 M** | 0,24 | 1,64 | 4,37 | 6,25 | 4,33 | 2,31 | 0,73 | 5,9 | 2,7 | 0,14 |
| **4 M** | 0,22 | 0,85 | 4,66 | 5,73 | 3,18 | 2,03 | 0,72 | 4,4 | 2,8 | 0,26 |
| **5 M** | 0,30 | 0,54 | 4,40 | 5,24 | 3,06 | 2,07 | 0,77 | 4,0 | 2,5 | 0,56 |
| **6 M** | 0,47 | 2,12 | 3,56 | 6,15 | 3,71 | 2,11 | 0,63 | 5,9 | 2,9 | 0,22 |
| **7 M** | 0,37 | 1,27 | 0,76 | 2,40 | 1,86 | 0,47 | 0,75 | 2,5 | 5,1 | 0,29 |
| **8 M** | 0,41 | 3,21 | 13,28 | 16,9 | 5,94 | 6,34 | 0,48 | 12,3 | 2,7 | 0,13 |
| **9 M** | 0,91 | 5,42 | 11,67 | 18,0 | 6,10 | 6,45 | 0,33 | 18,8 | 2,8 | 0,17 |
| **10 M** | 0,41 | 3,28 | 11,71 | 15,4 | 7,25 | 6,33 | 0,74 | 9,7 | 2,4 | 0,13 |
| **11 M** | 0,41 | 3,09 | 11,80 | 15,3 | 6,01 | 6,59 | 0,40 | 15,1 | 2,3 | 0,13 |
| **12 M** | 0,27 | 1,54 | 13,69 | 15,5 | 9,91 | 6,25 | 0,32 | 31,5 | 2,5 | 0,18 |
| **1 R** | 0,29 | 1,04 | 11,27 | 12,6 | 5,46 | 4,56 | 0,48 | 11,3 | 2,8 | 0,28 |
| **2 R** | 1,57 | 1,43 | 4,69 | 7,69 | 4,72 | 2,4 | 0,39 | 12,1 | 3,2 | 1,10 |
| **3 R** | 0,47 | 0,81 | 4,72 | 6,00 | 4,19 | 2,22 | 0,65 | 6,5 | 2,7 | 0,58 |
| **4 R** | 2,05 | 1,58 | 4,06 | 7,69 | 4,38 | 2,36 | 0,69 | 6,4 | 3,3 | 1,30 |
| **5 R** | 0,59 | 1,20 | 3,88 | 5,67 | 3,53 | 2,09 | 0,46 | 7,7 | 2,7 | 0,49 |
| **6 R** | 0,60 | 1,00 | 4,99 | 6,59 | 3,43 | 1,84 | 0,89 | 3,8 | 3,6 | 0,60 |
| **7 R** | 0,50 | 1,30 | 1,06 | 2,85 | 2,19 | 0,35 | 0,71 | 3,1 | 8,1 | 0,38 |
| **8 R** | 0,84 | 2,67 | 15,40 | 18,9 | 5,84 | 5,98 | 0,37 | 15,7 | 3,2 | 0,31 |
| **9 R** | 2,13 | 2,00 | 15,67 | 19,8 | 6,64 | 6,36 | 0,63 | 10,5 | 3,1 | 1,06 |
| **10 R** | 1,24 | 4,76 | 13,40 | 19,4 | 8,28 | 6,52 | 0,66 | 12,6 | 3,0 | 0,26 |
| **11 R** | 5,47 | 1,86 | 11,47 | 18,8 | 10,8 | 6,45 | 0,28 | 38,8 | 2,9 | 2,94 |
| **12 R** | 0,98 | 0,57 | 11,06 | 12,6 | 10,3 | 6,00 | 0,25 | 42,0 | 2,1 | 1,71 |
